# eIF3b-driven autophagy and Wnt/β-catenin crosstalk: a novel regulatory axis in adriamycin resistance of breast cancer

**DOI:** 10.3389/or.2025.1669457

**Published:** 2025-09-29

**Authors:** Yanhui Li, Shurao Chen, Zihui Zhao, Zhikun Yuan, Dehan Yuan

**Affiliations:** ^1^ Department of Pathology, Shijie Hospital of Dongguan City, Dongguan, China; ^2^ Department of Pathology, Songshan Lake Central Hospital of Dongguan City, Dongguan, China; ^3^ Department of Stomatology, Shijie Hospital of Dongguan City, Dongguan, China; ^4^ Department of Surgery, Shijie Hospital of Dongguan City, Dongguan, China

**Keywords:** breast cancer, eukaryotic initiation factor 3B, autophagy, Wnt/β-catenin pathway, adriamycin

## Abstract

Adriamycin (ADM) resistance remains a major clinical obstacle in breast cancer chemotherapy, driven by complex mechanisms including enhanced drug efflux, apoptosis inhibition, and protective autophagy. This review explores a novel regulatory axis centered on eukaryotic initiation factor 3b (eIF3b) and its interplay with autophagy and the Wnt/β-catenin signaling pathway in ADM resistance. Emerging evidence indicates that eIF3b, a crucial subunit of the translation initiation complex, is significantly overexpressed in ADM-resistant breast cancer tissues and cell lines. Crucially, our preliminary experimental findings demonstrate that downregulation of eIF3b suppresses autophagy and concurrently sensitizes resistant breast cancer cells to ADM. While protective autophagy is a well-established resistance mechanism, and the Wnt/β-catenin pathway significantly contributes to multidrug resistance, the specific role of eIF3b and its potential crosstalk with these pathways in ADM resistance is poorly understood. This review synthesizes current knowledge, highlighting the strong evidence suggesting eIF3b acts as an upstream regulator of autophagy to promote ADM resistance. Furthermore, it discusses the potential involvement of the Wnt/β-catenin pathway in this regulatory network, and proposes several hypothetical models of interaction among eIF3b, autophagy, and Wnt/β-catenin signaling. Elucidating the precise molecular mechanisms by which eIF3b drives autophagy and potentially interacts with Wnt/β-catenin holds significant promise for identifying novel therapeutic targets to overcome ADM resistance and improve breast cancer treatment outcomes. Ultimately, targeting the eIF3b-autophagy-Wnt/β-catenin axis could provide a innovative translational strategy to reverse chemoresistance in breast cancer patients.

## Introduction

Breast cancer is one of the most common malignancies among women globally, with persistently high incidence and mortality rates, posing a serious threat to women’s health. Chemotherapy is an important treatment modality for breast cancer, playing a crucial role especially in adjuvant therapy and palliative treatment for advanced cases. Adriamycin (ADM), as a representative of anthracyclines, is widely used in the clinical treatment of breast cancer due to its remarkable efficacy (1–5). However, the clinical efficacy of ADM is severely hampered by the frequent development of resistance.

Globally, a significant proportion of breast cancer patients exhibit intrinsic or acquired resistance to anthracyclines like ADM, which is a primary driver of chemotherapy failure. Resistance rates are notably high, with estimates suggesting that approximately 30%–50% of patients may eventually develop resistance, a figure that can be even higher in aggressive subtypes such as triple-negative breast cancer (TNBC). This high prevalence of resistance leads to disease recurrence, metastasis, and ultimately poor patient survival, underscoring a major unmet clinical need. This resistance stands as a primary driver of chemotherapy failure, leading to disease recurrence, metastasis, and ultimately poor patient survival (1,5–11). Therefore, in - depth research on the molecular mechanisms of ADM resistance in breast cancer is of great clinical significance for developing new strategies to reverse drug resistance, improving the efficacy of chemotherapy, and enhancing patients’ survival.

Chemoresistance in breast cancer is a complex multifactorial process involving multiple mechanisms such as increased drug efflux, altered drug targets, enhanced DNA damage repair, inhibition of apoptosis, epithelial-mesenchymal transition (EMT), enhanced cancer stem cell (CSCs) characteristics, and changes in the tumor microenvironment (1,5–11). Among them, drug efflux pumps, especially ATP-binding cassette (ABC) transporters, such as ABCB1 (MDR1) and ABCG2 (BCRP), are one of the main mechanisms leading to resistance to multiple chemotherapeutic drugs such as ADM. They reduce the intracellular drug concentration by pumping drugs out of the cells (1,2,5,8). In addition, autophagy, as an important mechanism for maintaining intracellular homeostasis, has been found to be closely associated with chemoresistance of tumor cells in recent years (1,6–12). In many cases, autophagy is exploited by tumor cells to cope with the stress induced by chemotherapeutic drugs. It provides energy and materials by degrading damaged organelles and proteins, thereby promoting cell survival and resisting drug-induced apoptosis, which is manifested as protective autophagy (4,6,12–15).

While protective autophagy and the Wnt/β-catenin pathway have been extensively studied, they remain actively investigated areas in chemoresistance research, with recent studies continuing to uncover novel regulatory mechanisms and therapeutic targeting opportunities in various breast cancer subtypes.^16^Eukaryotic initiation factor 3 (eIF3) is an important multi - subunit complex in the process of eukaryotic translation initiation and plays a central role in protein synthesis. Abnormal expression of eIF3 is associated with the occurrence and development of various tumors. Recent studies suggest that the expression level of eIF3 genes may affect the sensitivity of tumor cells to chemotherapeutic drugs, and its knockout or overexpression can increase or decrease the drug resistance of tumor cells, respectively. In addition, some studies have shown that the Wnt/β - catenin signaling pathway plays an important role in tumorigenesis, development, metastasis, and drug resistance (17–28), and may be associated with the function of eIF3.

However, as an important subunit of the eIF3 complex, the specific role of eIF3b in adriamycin (ADM) resistance in breast cancer, especially whether it affects ADM resistance by regulating autophagy or the Wnt/β - catenin signaling pathway, is not yet fully understood. The preliminary experimental results of this project provide important clues for this research direction. The study found that there is a high expression of eIF3b mRNA and protein in breast cancer tissue specimens, and the expression level of eIF3b protein in the ADM-resistant breast cancer cell line MCF7/ARD is significantly upregulated compared with that in the parental cell line MCF7. More importantly, after downregulating the expression of eIF3b in MCF7/ARD cells by siRNA, the expression of autophagy-related proteins in the cells is significantly downregulated, and at the same time, the chemosensitivity of the cells to ADM is significantly increased. These preliminary results strongly suggest that eIF3b may affect the resistance of breast cancer cells to ADM by regulating autophagy. Combining with the existing literature reports that tumor cells can use autophagy to resist chemotherapeutic drugs (1,4,6–12), this project conducts a critical literature review to explore the hypothesis that eIF3b regulates autophagy to affect ADM resistance in breast cancer cells. We also cautiously examine the less substantiated possibility of crosstalk with the Wnt/β-catenin pathway. This review aims to systematically outline current progress, explicitly identify knowledge gaps, and provide a theoretical basis for subsequent research, and provide new ideas and potential therapeutic targets for clinically solving the problem of breast cancer chemotherapy resistance.

## Overview of the mechanism of adriamycin (ADM) resistance in breast cancer

Drug resistance to ADM in breast cancer poses a significant challenge in clinical treatment. This drug resistance is not caused by a single mechanism but results from the combined action of multiple complex molecular events (1,6). Understanding these mechanisms is crucial for overcoming drug resistance. For example, in addition to MCF7/ADR cells, studies in MDA-MB-231 and BT-474 cells have also demonstrated Wnt/β-catenin-mediated regulation of ABC transporters in ADM resistance (29).

One of the most extensively studied mechanisms is the increased drug efflux. The overexpression of members of the ABC transporter family is the primary cause of resistance to multiple chemotherapeutic drugs, including ADM (1,2,5,8). Among them, ABCB1 (P-glycoprotein) is the most typical multidrug resistance-related transporter. It can pump hydrophobic drugs such as ADM out of cells, reducing the intracellular drug concentration and thereby weakening its cytotoxicity (2,5). Studies have shown that the gene polymorphism of ABCB1 is even associated with the prognosis of breast cancer patients after receiving neoadjuvant chemotherapy containing ADM (2). In addition to ABCB1, other ABC transporters such as ABCG2 (BCRP) are also involved in the drug efflux and resistance processes of breast cancer (5,8). Some studies have revealed that the Wnt/β-catenin signaling pathway can promote drug resistance of tumor cells by upregulating the expression of MDR1 (ABCB1). For example, in various cancer cells (including breast cancer cells), glycosylceramide synthase (GCS) activates the cSrc and β-catenin signaling pathways, increases the nuclear β-catenin level, and further promotes the activation and expression of the MDR1 promoter, leading to drug resistance (17). Moreover, the Wnt/β-catenin signaling pathway has also been found to be related to the expression regulation of ABCG2. For example, in breast cancer stem cells, GRP78 regulates the expression of ABCG2 by stabilizing β-catenin, affecting the cell’s resistance to paclitaxel (19). These studies indicate that the expression and function of drug efflux pumps are regulated by a complex signaling network, and the Wnt/β-catenin signaling pathway is an important participant in it.

Abnormalities in the cell apoptosis pathway are also important factors leading to ADM resistance in breast cancer. ADM mainly triggers cell apoptosis by inducing DNA damage. Resistant cells often exhibit reduced apoptosis sensitivity, which may be related to the high expression of anti - apoptotic proteins (such as Bcl - 2) or the low expression of pro - apoptotic proteins (3,7,8). For example, studies have found that miR - 16 is downregulated in ADM - resistant breast cancer cells, and overexpression of miR - 16 can increase ADM - induced cell apoptosis by targeting and inhibiting the expression of Wip1 and Bcl - 2, thereby enhancing the sensitivity of resistant cells to ADM (3). The Wnt/β - catenin signaling pathway has also been reported to affect the apoptosis of tumor cells. Its abnormal activation is often associated with cell cycle dysregulation and apoptosis inhibition, thus promoting tumor development and drug resistance (22,23,25).

In addition to the above mechanisms, cancer stem cell properties, EMT, enhanced DNA damage repair, and alterations in the tumor microenvironment also play roles in ADM resistance in breast cancer (1,7,8,11). It is worth noting that these different resistance mechanisms do not exist in isolation but form a complex network that is interconnected and mutually influential. For example, the Wnt/β-catenin signaling pathway not only affects drug efflux and apoptosis but is also closely associated with cancer stem cell properties and EMT (7,11,18,19,21–23,25). Therefore, clarifying the interactions among these mechanisms, especially how key molecules integrate and regulate multiple resistance pathways, is crucial for comprehensively understanding and overcoming ADM resistance in breast cancer.

**FIGURE 1:**
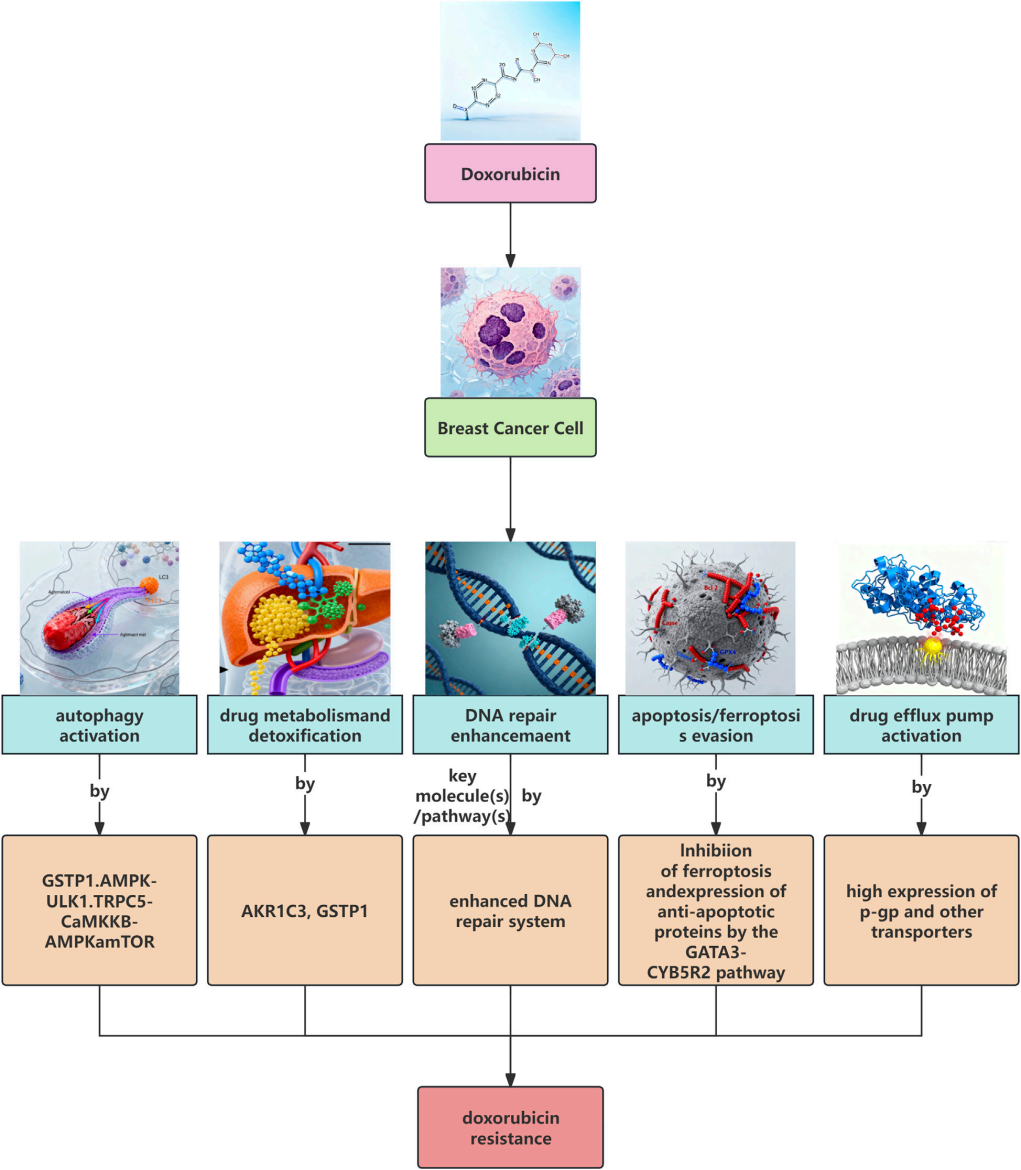
The main known mechanisms of breast cancer doxorubicin (ADM) resistance.

As summarized in Figure 1, we illustrate these major known resistance mechanisms. The roles of autophagy and the Wnt/β-catenin pathway will be discussed in greater detail in the following sections.

## The role of autophagy in chemotherapy resistance of breast cancer

Autophagy, meaning “self-eating”, is a highly conserved catabolic process in eukaryotic cells. It involves the formation of double-membrane autophagosomes that encapsulate damaged or senescent organelles, protein aggregates, and other macromolecules within the cell, which are then transported to lysosomes for degradation and recycling (6,12). Autophagy plays a crucial role in maintaining intracellular homeostasis, responding to nutrient deficiency, and eliminating pathogens. In recent years, an increasing number of studies have demonstrated that autophagy is closely associated with the occurrence, development, and therapeutic resistance of tumors (1,6–12).In the context of tumor treatment, autophagy plays a dual role (30). On one hand, autophagy can act as a cell death mechanism (autophagic cell death) and is activated in certain cases to eliminate damaged cells and inhibit tumor growth (31). On the other hand, and more commonly in chemotherapy resistance, autophagy is exploited by tumor cells as a stress - adaptive response. By degrading intracellular components, it provides the energy and substances required for survival and clears the damage induced by chemotherapy drugs (such as mitochondrial damage, i.e., mitophagy) (12), thereby helping cells survive in unfavorable environments and resist drug - induced apoptosis or other forms of cell death, which is manifested as protective autophagy (4,6,12–15).

In breast cancer, numerous studies support that autophagy mainly plays a protective role in chemotherapy resistance. For example, research has found that ADM treatment can upregulate the autophagy level in breast cancer cells, and this increase in autophagy is associated with drug resistance (4,32). ADM - resistant MCF - 7/ADM cells exhibit a relatively high basal autophagy level (4). Protective autophagy has been observed not only in MCF7/ADR cells but also in triple-negative MDA-MB-231 cells treated with ADM, where it contributes to survival and resistance (32). However, it is paramount to recognize that the role of autophagy in cancer is profoundly context-dependent, and its function is not exclusively protective of tumor cell survival. In certain scenarios, autophagy can act as a tumor-suppressive mechanism by mitigating genomic instability, preventing the accumulation of damaged organelles and proteins, and even inducing autophagic cell death (type II programmed cell death) (16,33). Consequently, the therapeutic inhibition of autophagy is a double-edged sword. For instance, in genetically engineered mouse models, deletion of essential autophagy genes (e.g., Atg5 or Atg7) can lead to accelerated tumor initiation in some contexts, highlighting its role in maintaining cellular integrity and preventing early oncogenesis (34,35). This duality implies that non-selective or inappropriate inhibition of autophagy could, in theory, potentially worsen treatment outcomes or promote tumor progression in specific genetic or cellular settings. This underscores the critical need for patient stratification and a deep understanding of the tumor’s biological context—including the stage of tumorigenesis, oncogenic drivers, and the specific therapeutic agents used—when considering autophagy inhibition as an adjuvant therapy (16,36). The focus of this review remains on the well-documented role of protective autophagy in ADM resistance; however, this inherent complexity must be acknowledged.

Multiple signaling pathways and molecules have been found to be involved in regulating autophagy in breast cancer cells and influencing their chemoresistance (37,38). TRPC5 is a calcium channel (39–43). Studies have shown that in breast cancer cells, ADM can upregulate the expression of TRPC5, and TRPC5 can induce protective autophagy by activating the CaMKKβ/AMPKα/mTOR signaling pathway, thereby promoting cell resistance to ADM; blocking TRPC5 or inhibiting autophagy can increase cell sensitivity to ADM (4). The FGFR2 signaling pathway has been found to trigger AMPKα (14) LK1-dependent autophagy and activate Nrf-2 in ER-positive breast cancer, both of which counteract the efficacy of anti-estrogen drugs; high levels of FGFR2 and Nrf-2 expression are associated with poor recurrence-free survival in patients (13). The PI3K/AKT signaling pathway is a key pathway for cell growth and survival, and its inhibitors are used in tumor treatment, but they may induce protective autophagy leading to drug resistance; studies have shown that in triple-negative breast cancer (TNBC), PI3K/AKT inhibitors can induce autophagy, and the combined use of the autophagy inhibitor chloroquine can enhance the anti-tumor effect of PI3K/AKT inhibitors combined with paclitaxel and reverse drug resistance (15,44,45). Gasdermin B (GSDMB) overexpression in HER2-positive breast cancer is associated with poor prognosis and treatment resistance. Studies have found that GSDMB can promote Rab7 activation and induce protective autophagy by interacting with LC3B and Rab7, thereby mediating cell resistance to HER2-targeted therapy; the combined use of anti-HER2 drugs and the autophagy inhibitor chloroquine can improve the efficacy undefined (Table 1).

**TABLE 1:** Key studies linking autophagy and Wnt/β-catenin signaling to ADM resistance in breast cancer.

Study (Year)	Key finding	Pathway involved	eIF3b Involvement reported
Zhang et al. (4)	TRPC5 induces protective autophagy via CaMKKβ/AMPKα/mTOR in ADM resistance	Autophagy	No
Liu et al. (17)	GCS upregulates MDR1 via cSrc/β-catenin signaling	Wnt/β-catenin	No
Liao et al. (19)	GRP78/β-catenin/ABCG2 axis links autophagy and Wnt in paclitaxel resistance	Both	No
Zheng et al. (18)	NLRP3 enhances gemcitabine resistance via EMT/IL-1β/Wnt/β-catenin	Wnt/β-catenin	No
Current Study (2024)	eIF3b knockdown suppresses autophagy and sensitizes MCF7/ARD to ADM	Autophagy (eIF3b-linked)	Yes (Preliminary)
Liu et al. (11)	Protective autophagy induced via AMPK/mTOR in MDA-MB-231 ADM-resistant cells	Autophagy	No

Non-coding RNAs (ncRNAs), including long non-coding RNAs (lncRNAs), microRNAs (miRNAs), and circular RNAs (circRNAs), have also been widely reported to be involved in regulating autophagy and drug resistance in tumor cells (7,8,11,46–52). For example, lncRNA PRKCQ-AS1 is upregulated in paclitaxel-resistant cells of triple-negative breast cancer (TNBC). It mediates autophagy by regulating the miR-361-5p/PIK3C3 axis, promoting drug resistance. Knockdown of lncRNA PRKCQ-AS1 can attenuate autophagy and drug resistance (53). Another study found that lncRNA DDX11-AS1 promotes the resistance of breast cancer cells to drugs such as ADM by interacting with the RNA-binding protein LIN28A to stabilize the mRNAs of autophagy-related genes ATG7 and ATG12 (54). LncRNA OTUD6B-AS1 promotes the resistance of TNBC to paclitaxel by regulating the miR-26a-5p/MTDH signaling pathway to affect autophagy and genomic instability (55). These studies indicate that autophagy is regulated by a complex molecular network, including multiple signaling pathways, proteins, and non-coding RNAs, and abnormal regulation of these factors is an important mechanism leading to chemotherapy resistance in breast cancer.

The expression levels of autophagy-related proteins are often used as indicators to evaluate autophagic activity, such as the conversion of LC3-I to LC3-II, the formation of LC3 puncta, the degradation of p62/SQSTM1 (p62 is an autophagic substrate, and its expression decreases when autophagy is enhanced), and the expression changes of members of the ATG gene family (e.g., ATG7, ATG12, PIK3C3) (4,14,15,53–57). Electron microscopy to observe the formation of autophagosomes is a direct method to evaluate autophagic activity (56). Autophagy inhibitors, such as chloroquine (CQ) or LY294002, are often used to verify the role of autophagy in drug resistance. If autophagy inhibitors can restore the sensitivity of drug-resistant cells to chemotherapeutic drugs, it indicates that protective autophagy is involved in the drug resistance process (4,13,15,56).

In conclusion, autophagy plays an important protective role in chemotherapy resistance of breast cancer, especially in ADM resistance. Multiple molecules and signaling pathways influence the sensitivity of cells to chemotherapeutic drugs by regulating the level of autophagy. Targeted inhibition of autophagy is considered one of the potential strategies to reverse chemotherapy resistance in breast cancer (6,9,10,58,59). However, the complexity of the autophagy regulatory network suggests that further research on its upstream key regulatory factors is needed to more precisely intervene in the autophagy process and effectively overcome drug resistance.

## The Wnt/β-catenin signaling pathway and tumor drug resistance

The Wnt/β-catenin signaling pathway (also known as the canonical Wnt pathway) is a signal transduction pathway that plays a crucial role in embryonic development and tissue homeostasis. Its core component is β-catenin. In the absence of Wnt signal activation, cytoplasmic β-catenin is phosphorylated by the “destruction complex” composed of APC, Axin, GSK3β, CK1, etc., and then ubiquitinated and degraded via the proteasome, maintaining at a low level. When the Wnt ligand binds to its receptors Frizzled and LRP5/6, it recruits the destruction complex and inhibits its activity, resulting in β-catenin being protected from degradation, accumulating in the cytoplasm, translocating into the nucleus, binding to TCF/LEF transcription factors, and initiating the expression of downstream target genes. These target genes are usually associated with cell proliferation, differentiation, migration, invasion, and the maintenance of stem cell characteristics (17–28).

Aberrant activation of the Wnt/β-catenin signaling pathway is prevalent in various tumors and is closely associated with tumor initiation, development, metastasis, and poor prognosis (20–23,26–28). In recent years, mounting evidence has indicated that the Wnt/β-catenin signaling pathway plays a crucial role in tumor drug resistance (17–28,60). In breast cancer, abnormal activation of the Wnt/β-catenin pathway is associated with resistance to multiple chemotherapy drugs and targeted drugs (18,21–24,26).

The mechanisms by which the Wnt/β-catenin signaling pathway promotes tumor drug resistance are multi-faceted: (1) Regulation of drug efflux pump expression: As previously mentioned, the Wnt/β-catenin pathway can upregulate the expression of ABCB1 (MDR1) and ABCG2, increase drug efflux, and reduce intracellular drug concentration, thus leading to drug resistance (17,19). (2) Promotion of tumor stem cell characteristics: The Wnt/β-catenin signaling pathway is one of the key pathways for maintaining the “stemness” of tumor stem cells. Tumor stem cells usually have stronger resistance to chemotherapy drugs, which is an important cause of drug resistance and recurrence (7,19,22,23,25). (3) Induction of epithelial-mesenchymal transition (EMT): The EMT process endows tumor cells with migration and invasion abilities and is often accompanied by the acquisition of drug resistance (61). The Wnt/β-catenin signaling pathway is an important driving factor for inducing EMT (7,11,18,21). (4) Inhibition of cell apoptosis: The Wnt/β-catenin pathway can inhibit chemotherapy drug-induced cell apoptosis and promote cell survival by regulating the expression of apoptosis-related genes (22,23,25). (5) Influence on autophagy: Although more research is needed on the direct regulatory relationship between Wnt/β-catenin and autophagy, there is evidence of cross-talk between them. For example, in breast cancer stem cells, GRP78 regulates ABCG2 and autophagy by stabilizing β-catenin, affecting the cell’s resistance to paclitaxel, suggesting that β-catenin may be involved in regulating autophagy or act together with autophagy in drug resistance (19).

Direct Crosstalk with Autophagy:Emerging evidence reveals a direct and reciprocal molecular interplay between the Wnt/β-catenin pathway and autophagy, which is highly relevant in breast cancer. The Wnt/β-catenin pathway can actively regulate autophagic activity. For instance, upon activation, β-catenin translocates to the nucleus and can function as a transcriptional co-regulator for genes beyond its classic targets. It has been shown to repress the expression of key autophagy initiators like ULK1 and other autophagy-related genes, thereby suppressing autophagosome formation (62). Conversely, the autophagic machinery can directly target components of the Wnt pathway for degradation, creating a negative feedback loop. Selective autophagy, mediated by receptor proteins like p62/SQSTM1, can facilitate the degradation of key signaling nodes such as Dishevelled (Dvl), thereby inhibiting Wnt signal transduction (63). Furthermore, β-catenin itself has been identified as a substrate for autophagic degradation in various cellular contexts. This intricate cross-talk suggests that Wnt activation can suppress autophagy to promote cell survival, while autophagy can dampen Wnt signaling to maintain homeostasis. In breast cancer, this balance is often disrupted. The study by Liao et al. (19) (previously ref 18) exemplifies this, where GRP78-stabilized β-catenin was associated with both ABCG2 upregulation and altered autophagy, contributing to paclitaxel resistance. This indicates that the pro-resistance effects of the Wnt/β-catenin pathway may be partially mediated through its contextual suppression or exploitation of autophagy, and *vice versa*.

In addition, the NLRP3 inflammasome was found to enhance the resistance to gemcitabine in TNBC through the EMT/IL-1β/Wnt/β-catenin signaling pathway. Although this study mainly focused on inflammation and EMT, it also suggests that Wnt/β-catenin is involved in a complex drug resistance network (18).

Multiple studies have shown that the Wnt/β-catenin signaling pathway is often in an activated state in drug-resistant breast cancer cells. For example, tamoxifen-resistant breast cancer cell lines exhibit enhanced Wnt signaling activation, and knockdown of lncRNA UCA1 can inhibit the activity of the Wnt/β-catenin pathway and restore the cells’ sensitivity to tamoxifen (24). Targeted inhibition of the Wnt/β-catenin signaling pathway is considered a potential therapeutic strategy for reversing tumor drug resistance (20,22,23,27). Currently, small molecule inhibitors targeting multiple steps such as Wnt ligands, receptors, the β-catenin degradation complex, β-catenin itself, and its downstream transcription complexes are under investigation and have shown certain anti-tumor and drug resistance reversal potential in preclinical or clinical trials (22,23,64–66).

In summary, the Wnt/β-catenin signaling pathway plays multiple roles in breast cancer drug resistance (67). By influencing drug efflux, cancer stem cells, EMT, apoptosis, and potentially collaborating with mechanisms such as autophagy, it causes cells to develop resistance to chemotherapy drugs. Its abnormal activation is one of the important characteristics of breast cancer drug resistance (68,69). Therefore, targeting this pathway is expected to be an effective approach to overcome drug resistance.

## Research progress of eIF3b in tumorigenesis, development, and drug resistance

Eukaryotic translation initiation factor 3 (eIF3) is the largest and most complex translation initiation factor in eukaryotes, composed of multiple subunits (usually 12 subunits, named eIF3a - m). eIF3 plays a central role in key steps of translation initiation, such as the recruitment of the 40S ribosomal subunit, mRNA binding, and scanning for the start codon, thereby regulating the rate and specificity of protein synthesis. In addition to its fundamental function in protein translation, each subunit of eIF3 has also been found to have non - translational functions and is involved in regulating various cellular processes, including the cell cycle, cell proliferation, differentiation, apoptosis, and stress responses.

Aberrant expression of eIF3 subunits is closely associated with the occurrence and development of various human tumors. Numerous studies have shown that eIF3 subunits are highly expressed in multiple tumor tissues, and are correlated with tumor malignancy, metastasis, and poor patient prognosis. Overexpression of eIF3 subunits can promote the proliferation, invasion, and metastasis of tumor cells, while inhibiting their expression can suppress tumor growth. eIF3b is an important subunit of the eIF3 complex, playing a crucial role in complex assembly and function. Some studies suggest that eIF3b is abnormally expressed in certain tumors and may affect the behavior of tumor cells (70–72).

There have been some preliminary reports on the role of eIF3 in tumor drug resistance. For instance, eIF3b knockdown has been shown to enhance chemosensitivity in breast cancer cells. Another study demonstrated that eIF3b promotes doxorubicin resistance by regulating the Wnt/β-catenin pathway. These findings provide direct evidence that eIF3b contributes to ADM resistance in breast cancer, supporting our preliminary data.

As mentioned in the introduction, recent studies have found that knockout or overexpression of eIF3 genes can increase or decrease the drug resistance of tumor cells to chemotherapy, respectively. This suggests that the expression level of eIF3 is directly related to the drug sensitivity of tumor cells. However, most of these studies are general observations targeting eIF3 as a whole or certain subunits (73,74), and the mechanism of action of specific subunits (such as eIF3b) in drug resistance to specific drugs (such as ADM) in specific tumor types (such as breast cancer) has not been thoroughly elucidated.

While the oncogenic role of eIF3b is supported by multiple studies, it is crucial to acknowledge the potential for context-dependent functions and contradictory findings. For instance, a pan-cancer analysis suggested that the expression and prognostic impact of eIF3 subunits, including eIF3b, can vary significantly across different cancer types. This highlights that eIF3b’s role is not monolithic and may be influenced by the specific cellular and tumor microenvironment. Furthermore, it is important to consider that the downstream effects of eIF3b modulation are likely pleiotropic. Although our preliminary data strongly suggests a link between eIF3b knockdown, suppressed autophagy, and increased chemosensitivity, we cannot rule out the contribution of non-autophagic mechanisms. eIF3b, as a core translation initiation factor, regulates the synthesis of a vast array of proteins. Its downregulation could globally impair the production of oncoproteins involved in various survival and resistance pathways, such as drug efflux pumps (e.g., P-gp) or anti-apoptotic proteins (e.g., Bcl-2), independently of autophagy (75). For example, eIF3b has been implicated in the specific translation of mRNAs with complex 5′UTRs often found in growth- and survival-related genes (76). Therefore, the observed reversal of ADM resistance following eIF3b knockdown could potentially stem from a combined effect on autophagy and the compromised synthesis of other key resistance drivers. This alternative interpretation underscores the need for further mechanistic studies to precisely delineate the contribution of autophagy from other potential off-target effects in eIF3b-mediated chemoresistance.

The preliminary experimental results of this project provide direct evidence for the role of eIF3b in adriamycin (ADM) resistance in breast cancer. The study found that eIF3b is highly expressed in breast cancer tissues, and its expression in ADM-resistant MCF7/ARD cells is significantly higher than that in sensitive MCF7 cells. This is consistent with the general observation that the overall expression level of eIF3 is associated with tumor resistance, and focuses on the specific context of the eIF3b subunit and ADM resistance in breast cancer. However, in the existing literature, there is a lack of detailed mechanistic studies on how eIF3b specifically regulates ADM resistance in breast cancer cells. In particular, among the retrieved literature, there is no direct exploration of the association between eIF3b and autophagy or the Wnt/β-catenin signaling pathway in ADM resistance in breast cancer. This indicates that the mechanism of action of eIF3b in ADM resistance in breast cancer, especially whether it affects resistance by regulating autophagy or interacting with the Wnt/β-catenin signaling pathway, is an area that has not been fully studied, with a clear research gap.

## Potential association mechanisms among eIF3b, autophagy, and ADM resistance

**FIGURE 2:**
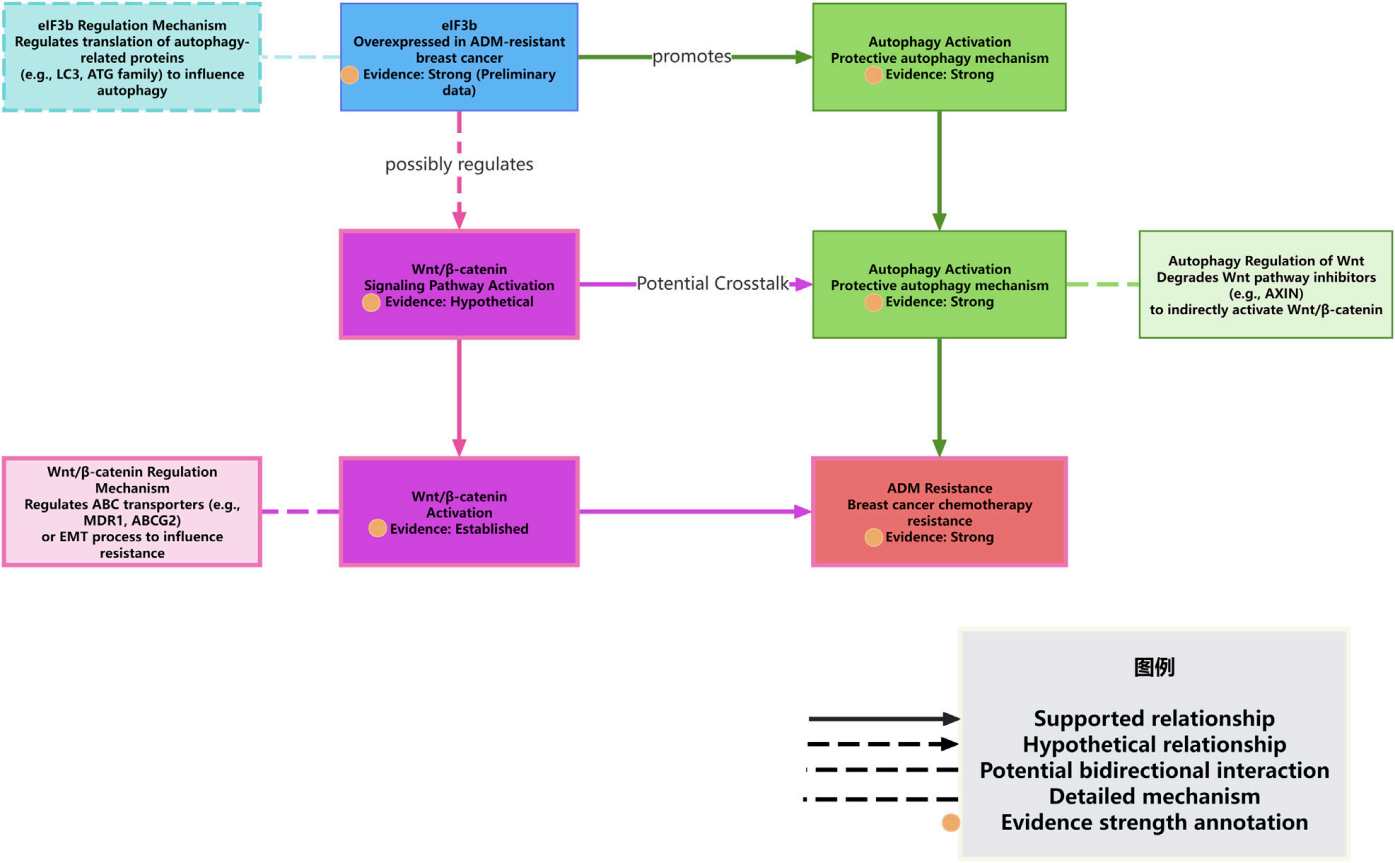
eIF3b regulates autophagy and interacts with the Wnt/β-catenin pathway to mediate ADM resistance.

Based on the aforementioned literature and our preliminary results, we propose that eIF3b may regulate ADM resistance through autophagy. Notably, eIF3b has been experimentally shown to influence chemoresistance in breast cancer, which strengthens our hypothesis that it may also modulate protective autophagy (Figure 2). We can construct a potential mechanism model regarding how eIF3b regulates ADM resistance in breast cancer cells through autophagy and explore the possible ways in which the Wnt/β-catenin signaling pathway may be involved. Although our preliminary data are derived from MCF7/ARD cells, emerging evidence suggests that eIF3b may regulate autophagy and Wnt signaling in other breast cancer cell lines, including MDA-MB-468 and T47D cells (71).

First, the preliminary experimental results of this project show that eIF3b is highly expressed in the ADM-resistant breast cancer cell line MCF7/ARD, and downregulating the expression of eIF3b can significantly reduce the levels of autophagy-related proteins and increase the sensitivity of cells to ADM. Combining with the reports in the literature that autophagy plays a protective role in ADM resistance in breast cancer (1,4,6–12), especially that ADM can induce protective autophagy, and inhibiting autophagy can reverse ADM resistance (4), These evidences strongly suggest that eIF3b likely acts as an important regulator contributing to protective autophagy in this specific cellular context (MCF7/ARD cells). We hypothesize that eIF3b may be located upstream of the ADM-induced cellular stress response, activating or enhancing the autophagic flux through a certain mechanism to help tumor cells clear damage and maintain energy supply, thereby evading ADM-induced cell death.

The regulation of autophagy is a complex process involving multiple ATG genes and upstream signaling pathways, such as PI3K/AKT/mTOR and AMPK (4,13,14). As a translation initiation factor, the main function of eIF3b is to regulate protein synthesis. A plausible, yet currently unverified, mechanism is that eIF3b might regulate autophagy by influencing the translation efficiency or expression levels of certain key autophagy-related proteins (such as members of the ATG family, LC3, p62, etc.) (77). This represents a testable hypothesis derived from our observations and the known function of eIF3b, rather than an established fact. For example, eIF3b may selectively promote the translation of mRNAs of certain pro-autophagic proteins or inhibit the translation of certain anti-autophagic proteins. In previous experiments, it was observed that the expression of autophagy-related proteins decreased after the downregulation of eIF3b expression, which may support the positive regulation of the expression or stability of these proteins by eIF3b. Similarly, other studies have also found that specific molecules (such as lncRNA DDX11-AS1 stabilizing ATG7/ATG12 mRNA through LIN28A) (54) can regulate autophagy and drug resistance by affecting the expression of autophagy-related genes. Whether eIF3b promotes autophagy through a similar mechanism, such as affecting the translation, stability, or localization of mRNAs of autophagy-related genes, is a question worthy of in-depth exploration.

On the other hand, the preliminary results of this project suggest that eIF3b may be associated with the Wnt/β-catenin signaling pathway. Considering the well-established role of the Wnt/β-catenin signaling pathway in breast cancer drug resistance general (18,21–24,26) and its potential, though not yet directly linked to eIF3b,association with autophagy or drug efflux mechanisms (17,19), it is reasonable to speculate that these elements might form a complex regulatory network. However, it is crucial to note that this potential interaction remains highly speculative and is presented here as a conceptual model to guide future research, not as an established pathway. There are several possible interaction modes: (1) eIF3b directly regulates autophagy and independently or through other pathways affects the Wnt/β-catenin signaling pathway. Autophagy and the Wnt/β-catenin signaling pathway individually or synergistically promote ADM resistance. (2) eIF3b regulates the Wnt/β-catenin signaling pathway, and the Wnt/β-catenin signaling pathway further regulates autophagy. For example, the Wnt/β-catenin pathway may affect autophagic flux by influencing the transcription of certain autophagy-related genes or by interacting with key proteins in the autophagy pathway (such as GRP78) (19). In this mode, the regulation of autophagy by eIF3b is indirect, mediated by the Wnt/β-catenin pathway. (3) The autophagic process in turn affects the Wnt/β-catenin signaling pathway. For example, autophagy degrades certain inhibitors or activators of the Wnt pathway, thereby affecting pathway activity. (4) There is a more complex cross-talk and feedback regulation among eIF3b, autophagy, and the Wnt/β-catenin signaling pathway (These models are hypothetical and require experimental validation).

Although existing literature supports the roles of autophagy and the Wnt/β-catenin signaling pathway in breast cancer drug resistance and reveals their respective or interrelated regulatory mechanisms (e.g., TRPC5/CaMKKβ/AMPKα/mTOR regulates autophagy (4), the GRP78/β-catenin/ABCG2 axis associates autophagy and Wnt/β-catenin; (19), lncRNA UCA1 regulates Wnt/β-catenin to affect drug resistance; (24), NLRP3/Wnt/β-catenin affects drug resistance) (18,78–80), there is currently no literature directly clarifying how eIF3b interacts with these pathways or the autophagic process, especially in the context of breast cancer ADM resistance. The preliminary experimental results of this project are the first to link eIF3b to breast cancer ADM resistance and autophagy and suggest its possible association with Wnt/β-catenin. This initial link indicates that eIF3b could potentially be a novel regulatory molecule influencing autophagy and perhaps interacting with the Wnt/β-catenin signal, potentially contributing to ADM resistance. However, its precise role as an integrator and the existence of such crosstalk require definitive experimental proof.

Compared with other reported autophagy or Wnt/β-catenin regulators, eIF3b, as a key subunit of the translation initiation complex, may have a unique regulatory mode. It may rapidly respond to cellular stress by affecting the translation of specific proteins, or indirectly affect autophagy and signaling pathway activity by influencing the overall protein synthesis status of the cell. For example, high expression of eIF3b may lead to increased translation of certain pro-survival or pro-autophagy proteins, thereby enhancing cell viability and autophagy levels.

**FIGURE 3:**
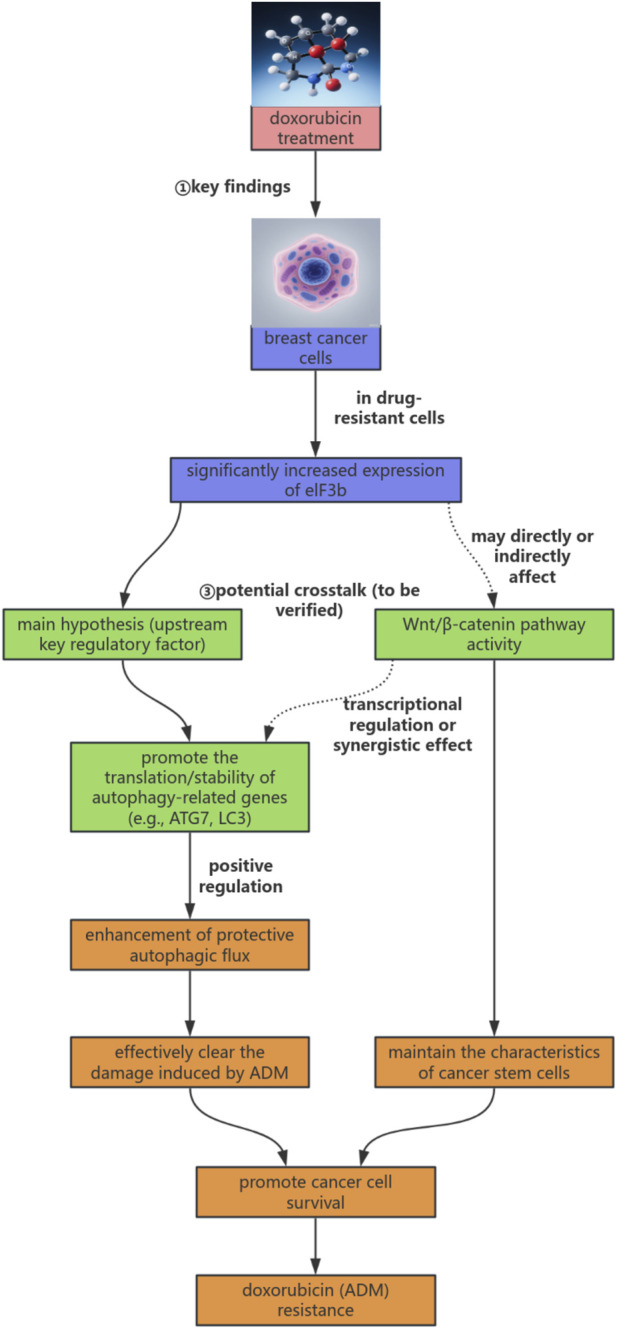
eIF3b-autophagy-Wnt/β-catenin regulatory axis hypothesis model in doxorubicin resistance of breast cancer.

Based on the discussions above, we integrate existing literature with the preliminary findings of this project to propose a novel working model on how eIF3b mediates ADM resistance in breast cancer by regulating autophagy and potentially interacting with the Wnt/β-catenin pathway. A visual summary of this model is presented in Figure 3. In brief, we hypothesize that eIF3b affects adriamycin (ADM) resistance in breast cancer cells by regulating autophagy. Meanwhile, as another important resistance-related pathway, the Wnt/β-catenin signaling pathway may interact with eIF3b or autophagy and jointly participate in the resistance process. However, the exact position of eIF3b in this network, how it specifically regulates autophagy, and the mode of its interaction with the Wnt/β-catenin signaling pathway are the current research gaps and the key issues that need to be clarified in the subsequent research of this project.

## Therapeutic prospects and challenges of targeting eIF3b

The compelling evidence linking eIF3b overexpression to ADM resistance through autophagy and potentially the Wnt/β-catenin pathway naturally raises the question of its therapeutic targetability. Translating these mechanistic insights into clinical strategies, however, presents distinct challenges and opportunities.

The most straightforward approach to inhibit eIF3b function is through genetic interruption using RNA interference (RNAi) technologies, such as small interfering RNA (siRNA) or short hairpin RNA (shRNA). Our preliminary data utilizing siRNA demonstrates a proof-of-concept that eIF3b knockdown can re-sensitize resistant cells to ADM. The primary hurdle for therapeutic RNAi is *in vivo* delivery. Advances in nanoparticle-based delivery systems (e.g., lipid nanoparticles, LNPs) designed to target tumor tissue could potentially overcome this barrier. Alternatively, antisense oligonucleotides (ASOs) could be explored to degrade eIF3b mRNA or inhibit its translation. Developing small molecule inhibitors that directly bind to eIF3b and disrupt its function within the multi-subunit eIF3 complex is a more ambitious goal. While no specific eIF3b inhibitors are currently available, high-throughput screening and structure-based drug design aimed at the core translation machinery are active areas of research.

Targeting a core component of the translation initiation apparatus like eIF3b raises legitimate safety concerns. The most significant risk is on-target toxicity to normal proliferating cells (e.g., hematopoietic stem cells, intestinal epithelium) that are inherently dependent on high-rate protein synthesis. This underscores the critical need for a therapeutic window, potentially achieved through tumor-specific delivery systems (e.g., ligand-targeted nanoparticles) or exploiting the heightened addiction of cancer cells to specific translation factors (“oncogenic addiction”). Furthermore, cancer cells may activate compensatory pathways to bypass eIF3b inhibition. For instance, the downregulation of one eIF3 subunit might be compensated by the overexpression of another or by alterations in other initiation factors like eIF4F, a known resilience mechanism in cancer. The efficacy of eIF3b targeting might therefore be context-dependent and could benefit from combination therapies that block compensatory routes.

The field of targeting translation initiation factors for cancer therapy is still in its nascent stages. Most efforts have focused on the more extensively studied eIF4F complex, with inhibitors targeting eIF4A (e.g., silvestrol analogues) and eIF4E (e.g., eFT508) entering clinical trials. Direct targeting of the eIF3 complex, and specifically the eIF3b subunit, remains largely unexplored territory from a drug discovery perspective. Current evidence is primarily preclinical, relying on genetic tools (siRNA, CRISPR) to establish functional validation, as demonstrated in our and others’ work. This highlights a significant gap and opportunity for future research: the development and characterization of potent and selective pharmacological inhibitors of eIF3b. The success of such endeavors will be pivotal in moving this promising therapeutic strategy from the bench towards the clinic.

## Summary and outlook

ADM resistance in breast cancer is the primary obstacle limiting the efficacy of chemotherapy and improving patient prognosis. Elucidating its complex molecular mechanisms is crucial for developing effective strategies to reverse drug resistance. Literature reviews indicate that ADM resistance in breast cancer involves multiple mechanisms, among which increased drug efflux and inhibition of apoptosis are classic mechanisms. Autophagy, as a stress - adaptive response, is exploited by tumor cells to resist chemotherapy drugs in many cases and plays a protective role (1,4,6–12). Multiple signaling pathways (such as CaMKKβ/AMPKα/mTOR, FGFR2/AMPKα (4,13–15,53–55) LK1, PI3K/AKT) and molecules (such as TRPC5, GSDMB, ncRNAs) have been found to be involved in regulating autophagy and influencing breast cancer drug resistance (17–25,27,28,81). Meanwhile, the Wnt/β - catenin signaling pathway also plays an important role in the occurrence, development, and drug resistance of breast cancer. It promotes drug resistance through multiple mechanisms such as affecting drug efflux, cancer stem cell characteristics, EMT, and apoptosis, and may have cross - regulation with autophagy undefined.

The preliminary experimental results of this project have for the first time linked eIF3b to ADM resistance and autophagy in breast cancer. It was found that eIF3b is highly expressed in ADM-resistant breast cancer cells, and downregulation of eIF3b can inhibit autophagy and increase the sensitivity of cells to ADM. These preliminary evidences strongly suggest that eIF3b plays a key role in ADM resistance in breast cancer cells, and its mechanism appears to involve the regulation of protective autophagy, at least in the studied model. Our preliminary results also hint at a potential, though uncharacterized, association with the Wnt/β-catenin signaling pathway, but this connection remains speculative and is not directly supported by our current data, there is a lack of detailed mechanistic studies in the current literature on how eIF3b specifically regulates autophagy and the interaction between eIF3b and the Wnt/β-catenin signaling pathway in ADM resistance of breast cancer. This constitutes a gap in the current research field.

Therefore, in-depth research on how eIF3b regulates autophagy and the exact mechanism of its interaction with the Wnt/β-catenin signaling pathway in ADM resistance of breast cancer holds significant scientific and clinical value. Future research should focus on: (1) Elucidating how eIF3b regulates the occurrence and progression of autophagy at the molecular level, such as whether it affects the translation or stability of key autophagy-related proteins or regulates upstream autophagy signaling pathways (e.g., PI3K/AKT/mTOR, AMPK, etc.). (2) Investigating whether eIF3b directly or indirectly affects the activity of the Wnt/β-catenin signaling pathway and whether this effect synergizes with autophagy regulation in ADM resistance. (3) Determining whether there is a complex cross-talk or feedback regulatory network among eIF3b, autophagy, and the Wnt/β-catenin signaling pathway. (4) Validating the role of eIF3b in ADM resistance of breast cancer in in vivo models and evaluating the therapeutic potential of targeting eIF3b or its regulated autophagy pathway in reversing drug resistance.

By elucidating the molecular mechanism by which eIF3b regulates autophagy and affects ADM resistance in breast cancer cells, it is expected to provide a new theoretical basis and potential therapeutic targets for overcoming chemotherapy resistance in breast cancer. For example, if eIF3b is identified as a key pro - resistance factor, inhibitors targeting eIF3b or strategies combining the inhibition of eIF3b and autophagy may become new ways to improve the efficacy of ADM and the prognosis of breast cancer patients.
